# Recent advances in the molecular biology of the protist parasite *Trichomonas vaginalis*

**DOI:** 10.12703/r/10-26

**Published:** 2021-03-04

**Authors:** David Leitsch

**Affiliations:** 1 Department of Specific Prophylaxis and Tropical Medicine, Medical University of Vienna, Vienna, Austria

**Keywords:** Trichomonas vaginalis, Sexually Transmitted Diseases, Microbiome

## Abstract

*Trichomonas vaginalis* is an anaerobic/microaerophilic protist parasite which causes trichomoniasis, one of the most prevalent sexually transmitted diseases worldwide. *T*. *vaginalis* not only is important as a human pathogen but also is of great biological interest because of its peculiar cell biology and metabolism, in earlier times fostering the erroneous notion that this microorganism is at the root of eukaryotic evolution. This review summarizes the major advances in the last five years in the *T*. *vaginalis* field with regard to genetics, molecular biology, ecology, and pathogenicity of the parasite.

## Introduction

The protist parasite *Trichomonas vaginalis* is the causative agent of trichomoniasis, one of the most prevalent sexually transmitted diseases worldwide. In the most recent estimate by the World Health Organization^1^, 110 million infections worldwide were ascribed to *T*. *vaginalis* in 2016; that is somewhat less than ascribed to chlamydia but much more than to gonorrhea and syphilis. Symptoms of trichomoniasis are usually more severe in women than in men and affect the vagina, the cervix, or the urethra or a combination of these. Vaginal pruritus and odorous vaginal discharge are the most common sequelae, but severe inflammation of the cervix can also occur. In men, *T*. *vaginalis* can cause urethritis and prostatitis, albeit with lower frequency. Trichomoniasis can persist for years and sour the life of those infected, but it also increases the risk for adverse pregnancy outcomes and the risk of acquiring HIV^2^. It is now firmly established that underlying trichomoniasis increases the risk to acquire HIV by 1.5- to 2-fold^3^. This poses serious problems in countries where both pathogens are highly endemic (for example, in the southern part of Africa). *T*. *vaginalis* has also been suggested to predispose for cervical cancer and prostate cancer, although there is far less support for this supposed link^4–6^ than with HIV.

However, *T*. *vaginalis* not only is of medical importance but also is a fascinating microorganism in its own right. Once considered a primordial eukaryote because of its anaerobic metabolism and the absence of mitochondria, it is now known to host hydrogenosomes^7^, hydrogen gas–producing organelles derived from mitochondria. Furthermore, *T*. *vaginalis*, like other trichomonads, has a huge haploid genome of 160 Mb which contains a very large number of transposable elements (TEs)^8^. *T*. *vaginalis* is commonly believed to be asexual and to exist only as a trophozoite-stage flagellate, but the recent discovery of meiosis-specific genes^9^, amongst other features typical of sexual organisms, and of a cyst-like stage with a cell wall^10^ might lead to a revaluation of these tenets.

The present overview focuses on the major scientific advances in the *T*. *vaginalis* field in the last five years and covers genetics, biochemistry, cell biology, ecology, and infection biology of the parasite. Drug testing and resistance will also be discussed. However, more clinical aspects of trichomoniasis, such as epidemiology, diagnosis, and management, were surveyed recently in another review published at F1000 Research^11^ and are not included here.

## Genome and gene expression

As compared with other protists, *T*. *vaginalis* has an enormously large genome of 160 Mb in length^12^, which encodes about 60,000 protein-coding genes. A surprisingly large portion of the genome consists of TEs^13,14^, and as many as 40,000 TEs come from several families. This has slowed down efforts to assemble the genome because of the immensely high number of sequence repeats. Still, the availability of the genome sequence, if not fully assembled, has allowed substantial scientific advances on *T*. *vaginalis* evolution on the one hand and on its genetic machinery on the other.

Strikingly, *T*. *vaginalis* has a predicted number of only 62 introns. A detailed study on introns in *T*. *vaginalis* confirmed the existence of 32 genes with introns, whereas 18 candidates were found to be non-functional as they were not removed from the respective transcripts^15^. Eleven new introns which group into two different types with regard to sequence and splice motifs were identified in this study. Transcription of protein-encoding genes is commonly initiated at an initiator element^16^ or two alternative promoter elements, M3 and M5, respectively^17^. Interestingly, the M3 element resembles a Myb recoginition element and was found to be bound by a novel transcription factor with a Myb-like DNA-binding domain^17^. Surprisingly, however, a TATA box was found to be missing from promoters of protein-encoding genes. *T*. *vaginalis* nonetheless does encode two TATA box-binding proteins (TBPs) which bind to initiator-binding protein 39 (IBP39)^16^ and to DNA as determined in electrophoretic mobility shift assays^18^. However, it seems that binding to DNA by these *T*. *vaginalis* TBPs is unspecific^19^. Instead, they might have a role in the transcription of spliceosomal U6 snRNA. Also of interest is the mRNA capping apparatus in *T*. *vaginalis* which is structurally similar to its counterparts in metazoans and plants rather than to those in other protists^20^.

Transcription rates not only are determined by sequences proximal to the transcription start site but also are dependent on epigenetic regulation. Consequently, epigenetic regulation in *T*. *vaginalis* was addressed in a number of studies. N6-methyladenine (6mA) was found to be the major DNA methylation mark in *T*. *vaginalis*^21^. It occurs frequently in intergenic regions (in 94% of all sequenced methylated DNA stretches) and probably localizes to chromatin loops, indicating a role of 6mA in the regulation of gene expression. Also, histone acetylation was identified as an important modulator of gene expression in two independent studies^22,23^. Indeed, *T*. *vaginalis* has a large arsenal of histone deacetylases (HDACs) of the Sir2 type and of histone acetyl transferases^22^. Methylation and acetylation of histone 3 (H3K27Ac and H3K4Me3, respectively) near transcription initiation sites were found to be positively correlated with transcription rate, and the HDAC inhibitor trichostatin A was found to strongly affect gene expression^23^. Independently of the transcription rate, mRNA levels can also be regulated, a process termed RNA interference (RNAi). For RNAi, the presence of small RNAs (sRNAs), which are complementary to their target mRNA and recruit the so-called RNA-induced silencing complex, is a prerequisite. Importantly, two Argonaute proteins (*T*. *vaginalis* AGO1 and 2), which constitute central components of this complex, have been found in the genome^14^. Furthermore, a large number of sRNAs of the PIWI domain–interacting type (piRNA) were found. Arguably, *T*. *vaginalis* AGO1 and 2 interact with sequence-specific piRNA to degrade transposase mRNAs as encoded by myriads of TEs in the *T*. *vaginalis* genome^14^.

The last step of expression of protein-coding genes (that is, translation) has also been studied in recent years. Translation efficiency of transcripts was found to be tuned by stretches of low sequence complexity at the 5′ end of the mRNA, followed by a highly structured region more downstream^24^. The newly introduced iLOV fluorescent protein^25^ was used as a marker to gauge the effect of the prospective regulatory sequences on protein expression.

In addition to the fundamental research on *T*. *vaginalis* genetics as stated above, the repertoire of genetic tools in this parasite was refined and enlarged. In a comprehensive study, reference genes for quantitative polymerase chain reaction studies were evaluated^26^. Importantly, the frequently chosen glyceraldehyde 3-phosphate dehydrogenase (*GAPDH*) gene was identified as highly unreliable when exposing *T. vaginalis* to various conditions due to the strongly variable expression levels observed. In contrast, the genes for DNA topisomerase 2, α-tubulin, and actin were found to be much more suitable; the best dual combination was DNA topisomerase 2 and α-tubulin. Hopefully, these results will be taken into account in future studies on gene expression in *T*. *vaginalis*. Furthermore, RNAi was applied successfully for the first time in *T*. *vaginalis*^27^, although downregulation of the respective transcripts was rather low (33–72% at the most), which might be insufficient for many scientific questions. As a word of caution, the feasibility of the method still awaits confirmation from other researchers in the field. In the meantime, gene knockouts using CRISPR/Cas-9 technology might prove highly instrumental. In a pilot study^28^, CRISPR/Cas-9 was successfully applied to knock out the non-essential genes for ferredoxin-1 and migration inhibitory factor. Importantly, the efficiency of the necessary transfection procedures was highly improved when a newly developed nucleofection protocol was applied.

## Cell biology and biochemistry

*T*. *vaginalis* diverges strongly from most other protists by having an anaerobic metabolism and by hosting an unusual organelle, the hydrogenosome. These issues have attracted considerable interest throughout the last 50 years, and the last five years have yielded further important insights into the physiology of this parasite.

The hydrogenosome was originally believed to have evolved from a bacterial endosymbiont in a trichomonad progenitor but is now known to derive from mitochondria^29^. Many biological processes relating to hydrogenosomal function, however, remained incompletely understood in the past. Protein import into hydrogenosomes, for example, is of pivotal importance as no protein translation takes place within the organelle. Some hydrogenosomal proteins have an N-terminal targeting sequence (NTS) but others do not^30,31^. Moreover, in many cases, the NTS seems to be dispensable for protein import, and even ectopic import into yeast mitochondria without NTS is possible^31,32^. This indicates that predominantly internal signals earmark certain proteins for import into hydrogenosomes. In the case of tail-anchored (TA) proteins which localize to the *T*. *vaginalis* hydrogenosome^33^, the responsible amino acid sequences have been studied in more detail: in the C-terminal region next to the trans-membrane domain of TA proteins, the net charge must be positive to ensure reliable import. The translocase of the outer membrane (TOM) complex is mainly responsible for conducting protein import into the hydrogenosome^34^. The translocation channel is mainly formed by TvTOM40-2 which is divergent from TOM40 proteins from other eukaryotes but which nevertheless can partially complement for TOM40 mutations in yeast^34^. Despite its apparent simplicity, the protein import machinery of the hydrogenosome is highly effective. In recent decades, there was an intense discussion about whether certain highly expressed hydrogenosomal proteins such as pyruvate:ferredoxin oxidoreductase (PFOR), malic enzyme, and succinyl-coA synthetase (SCS) are also trafficked to the cell surface to act there as adhesin proteins, facilitating adhesion to host epithelium. This issue has now been conclusively settled by a recent study which showed that these enzymes are exclusively trafficked into the hydrogenosomes^35^. However, trafficking of proteins to membrane compartments in general (that is, to destinations including the hydrogenosome but not being restricted to it) seems to have a different underlying mechanism as demonstrated for the cyclophilins TvCyP1 and TvCyP2^36^.

The major hydrogenosomal pathway deploys PFOR, SCS, malic enzyme, and hydrogenase for the breakdown of pyruvate and malate to carbon dioxide and hydrogen gas. But also pathways for amino acid catabolism are hosted in the organelle. These become more important under glucose restriction, especially the arginine dihydrolase pathway^37^. The arginine dehydrolase pathway also indirectly leads to the formation of nitric oxide (NO) levels and this has a stabilizing effect on hydrogenosomal membranes under glucose restriction^38^. Several hydrogenosomal enzymes (for example, PFOR and hydrogenase) are sensitive to oxygen and its derivatives such as hydrogen peroxide and superoxide radical anion and need to be protected by appropriate antioxidant enzymes (reviewed in 39). The most recently discovered antioxidant enzyme of the hydrogenosome is the osmotically inducible protein C (OsmC), which detoxifies peroxides after receiving electrons from lipoate via the glycine decarboxylase L and H proteins^40^.

Pyruvate and malate are broken down in the hydrogenosome but derive from glycolysis taking place in the cytosol. Glucose, in turn, is obtained mostly from the breakdown of intracellular glycogen^41^ or of glycogen from the vaginal environment. *T*. *vaginalis* secretes several glycosidases^42^ (including most notably β-amylase^43,44^), which break down glycogen to maltose. Maltose is further broken down to glucose by another glucosidase^45^, followed by glucose import into the cell. Of course, carbohydrate uptake is not the only form of nutrient uptake *T*. *vaginalis* is capable of. The hydrolysis of nucleotides from the host by *T*. *vaginalis* ectonucleoside triphosphate diphosphohydrolase (E-NTPDase) and the subsequent uptake of the resulting nucleosides have been studied in a suite of studies in recent years^46,47^.

## *Trichomonas vaginalis* as a member of the vaginal microbiome

The main habitat of *T*. *vaginalis*, the human vagina, accommodates a highly complex microbiome^48^ which strongly influences the chances of *T*. *vaginalis* to successfully colonize its host. The microbiota of the vagina are roughly grouped into five community state types (CSTs) of which four (CST-I, -II, -III, and -V) are dominated by lactobacilli, whereas CST-IV is dominated by anaerobic bacteria such as *Gardnerella vaginalis* and mollicutes such as *Mycoplasma* spp.^49^. Lactobacilli preserve an acidic environment (pH ~4.5), whereas CST-IV leads to a higher vaginal pH. *T*. *vaginalis* is predominantly associated with CST-IV^50^. The presence of certain anaerobic bacteria (that is, *Prevotella amnii* and *Sneathia sanguinegens*) is positively correlated with the acquisition of *T*. *vaginalis*^51^. Interestingly, representatives of the CST-IV microbiome enhance adherence of *T*. *vaginalis* to epithelial cells through formation of a biofilm^52^ and both can cooperatively compromise the integrity of the vaginal epithelium by disrupting intercellular tight junctions^53^. In contrast, lactobacilli clearly antagonize *T*. *vaginalis*. In a revelatory study, *Lactobacillus gasseri* strain ATCC 9857^54^ was shown to strongly inhibit adhesion of *T*. *vaginalis* to host cells in a contact-dependent manner. In fact, *L*. *gasseri* ATCC 9857 could even displace already-adhering trichomonads from vaginal epithelial cells. Arguably, aggregation-promoting factor 2 (APF-2) as encoded by the lactobacilli is responsible for this remarkable capability. *T*. *vaginalis*, however, is by no means defenseless. It expresses nine peptidoglycan hydrolases of the NlpC/P60 family which can kill bacteria^55^. To date, these enzymes have been tested only on *Escherichia coli* and it will be interesting to learn more about their activity against vaginal bacteria.

Even if most details of the interplay of *T*. *vaginalis* with the microbiota of the vagina remain to be discovered, the association of *T*. *vaginalis* with mollicutes, predominantly with *Mycoplasma* spp., has been studied in considerable detail. The proportion of *T*. *vaginalis* isolates harboring *Mycoplasma hominis* varies strongly depending on geographic origin but usually is substantial and can be even higher than 80%^56^. Thus, coinfections with *T*. *vaginalis* and *M*. *hominis* are very common and therefore should be taken into account by all means when speaking of trichomoniasis. The interactions of *T*. *vaginalis* and *M*. *hominis* are mutually beneficial. Most importantly, the growth rate of *T*. *vaginalis* is greatly enhanced (that is, by 20%) if intracellular *M*. *hominis* is present^57^. This is probably due to the arginine dihydrolase (ADH) pathway which is shared by the two microorganisms and which leads to the production of more ATP when L-arginine is present as a substrate. Indeed, intracellular *M*. *hominis* was shown to enhance ATP production substantially after supplementation with L-arginine whereas in symbiont-free *T*. *vaginalis* supplementation of arginine had only a minimal effect. This also has implications for the host defense because NO production by immune cells is greatly diminished if L-arginine is scavenged by the *T*. *vaginalis*/*M*. *hominis* consortium. There are probably many more interactions, and there is some evidence that *M*. *hominis* can alter gene expression in *T*. *vaginalis* to a certain extent^58^. Like *T*. *vaginalis*, *M*. *hominis* has been linked to adverse pregnancy outcomes^59^. There is an indication that the cohabitation with *T*. *vaginalis* promotes this, especially when *T*. *vaginalis* is eliminated by metronidazole treatment and liberated *M*. *hominis* infects host tissue^60,61^. In addition to *M*. *hominis*, another *Mycoplasma* species is closely associated with *T*. *vaginalis*: *Mycoplasma girerdii*. This species was discovered only recently^62^, possibly because it might exist merely as an intracellular symbiont of *T*. *vaginalis*. A recent study, however, casts doubt on this notion^51^. In any case, *M*. *girerdii* is at least as common as *M*. *hominis*^63^ but its pathogenic potential remains to be elucidated.

In addition to hosting bacterial symbionts, *T*. *vaginalis* hosts *T*. *vaginalis* virus (TVV), which is grouped into four strains (TVV1–4) and belongs to the family of Totiviridae^64^. Little is known about the life cycle of TVV but it has been suggested to exacerbate trichomoniasis by enhancing the immune response^65^, and a recent study showed that the presence of TVV mitigates the response of vaginal epithelial cells to trichomonads^66^. However, another recent study of 355 *T*. *vaginalis* isolates, of which 40% hosted TVV, did not find any association of TVV and clinical symptoms^67^.

## Pathogenesis of trichomoniasis

After a long period of scientific neglect, the pathogenesis of trichomoniasis is now finally receiving the attention it deserves. In recent years, pertinent studies have been forthcoming in increasing numbers, covering numerous aspects ranging from host response to parasite virulence factors (reviewed in 68).

Arguably the most significant response of the host to *T*. *vaginalis* is the production of cytokines by immune cells at the site of infection. Mainly interleukin 1 (IL-1), IL-6, IL-8, and IL-17^69–71^ are induced and this is characteristic of a pro-inflammatory response. Interestingly, the cytokine profile was influenced by the presence of the *M*. *hominis* as IL-1 and IL-6 levels were several-fold higher after exposure to *T*. *vaginalis* G3 with the endosymbiont as compared with the same strain without^69^. Notably, IL-6 was reported to induce polarization of THP-1–derived macrophages into M2-type macrophages^72^ and IL-1 production is linked to activation of the NLRP3 inflammasome in macrophages resulting in processing of precursor IL-1β to bioactive IL-1^73^. IL-1, in turn, can induce pyroptotic cell death in macrophages^73^. The host cell response to *T*. *vaginalis* further centers on Toll-like receptor 2 (TLR2), whose expression is also triggered by *T*. *vaginalis*^71,74^. Consequently, in TLR2^−/−^ mouse macrophages, immune mediators such as p38, ERK, and p65 NF-κB were found to be phosphorylated to a lesser extent after stimulation with *T*. *vaginalis*^75^. In contrast to these observations, intraepithelial dendritic cells and regulatory T cells were shown to react to exposure with *T*. *vaginalis* or one of its major antigens, actinin-2, with the expression of IL-10, which is an anti-inflammatory cytokine^75^. Cytokine production, however, is not restricted to the human host as *T*. *vaginalis* also produces cytokines to ensure its survival. The parasite secretes a homologue of human macrophage migration inhibition factor (TvMIF), which increases survivability under serum starvation several-fold^76^. Serum contains several essential nutrients for *T*. *vaginalis*, such as amino acids, lipids, and precursors of nucleotides. *T*. *vaginalis* also secretes leukotriene B4 (LTB4), which induces exocytotic degranulation in mast cells, thereby promoting tissue inflammation^77^.

*T*. *vaginalis* interacts with the host tissue by shedding extracellular vesicles (EVs) carrying cargo which promotes the infection process (thoroughly reviewed in 78). Two types of *T*. *vaginalis* EVs have been identified: exosomes (50–150 nm)^79^ and microvesicles^80^, which are considerably larger (100–1000 nm). The former derive from intracellular multivesicular bodies whereas the latter are shed from the cell membrane. The vesicles contain protein factors involved in mediating adherence, such as tetraspanins or BspA-domain proteins, and arguably in tuning the host’s response. Notably, the content of the small vesicles was found to be altered in the presence of TVV^66^. In addition to proteins, EVs contain RNA^81^; that is, mainly tRNA fragments or tRNA halves (tsRNA), respectively, which have also been described to be part of the cargo in EVs of trypanosomatid parasites^82,83^. The precise role of tsRNA in host–parasite interactions, however, remains to be elucidated. Finally, the uptake of exosomes by host cells is mediated by 4-α-glucanotransferase (Tv4AGT) on the EV surface which binds to heparan sulfate of host cell surface proteoglycans^84^.

Adherence of *T*. *vaginalis* to host epithelium is indeed a key event in trichomoniasis. In this process, the host’s surface proteins galectin-1^85^ and -3^86^ have an essential role by binding to lipoglycan (LG) on the parasite’s surface^85^. Host galectins are also instrumental in dimming the host response to *T*. *vaginalis*, and TvLG binding can further tune this^86^. However, numerous other cell surface–associated factors, such as a novel cadherin-like protein^87^, actinin-2^88^, and triosephosphate isomerase^89^, promote adherence of *T*. *vaginalis* to host epithelium. The last of these is a glycolytic protein which can also be associated with the parasite’s cell surface and bind to fibronectin and laminin. Two other groups of proteins, the BspA and Pmp domain-containing proteins, seem to enhance adherence^90^, and the former have been found to be transported to the host in EVs^79^. Finally, palmitoylation of proteins was found to positively affect adherence of *T*. *vaginalis* to host cells^91^.

Eventually, adherence is followed by cell lysis. Damage to host cells can be inflicted by parasite proteases such as metalloprotease TvMP50^92^ or cysteine proteinase 2 (CP2)^93,94^, and antibody treatment against either of these factors greatly diminished cytotoxicity as exerted by *T*. *vaginalis*. Interestingly, *T*. *vaginalis* cysteine proteases do also degrade the anion channel CFTR on the host cell surface, leading to elevated intracellular Cl^−^ concentrations and induction of NF-κB signaling^95^. Another protease, the rhomboid protease TvROM1, exerts an indirect effect by cleaving *T*. *vaginalis* substrate proteins, resulting in enhanced attachment and damage to host cells^96^. Just recently, saponin-like pore-forming proteins (TvSaplips) were identified in the *T*. *vaginalis* genome^97^ and one of these, TvSaplip12, was expressed and characterized. TvSaplip12 is strongly upregulated upon contact with host cells and has a strong lytic activity against bacteria and HeLa cells. Consequently, it has been proposed to act as a *Trichopore*, in accordance with Amoebapore in *Entamoeba histolytica*^98^. To summarize, the picture of pathogenesis of trichomoniasis is still incomplete but the gaps are being filled at an increasing pace.

## Anti-trichomonadal drugs: established and experimental

Throughout the last six decades, the 5-nitroimidazole drug metronidazole has remained the mainstay of anti-trichomonadal chemotherapy^99^, although resistance has become an increasingly worrying issue. Clinical metronidazole resistance is a complex phenomenon affecting numerous enzymatic pathways in *T*. *vaginalis*^99^. In a large-scale study on gene expression in metronidazole-sensitive and -resistant strains^13^, several genes were found to be differentially expressed in three metronidazole-resistant strains assayed. Flavin reductase 1 (FR1), an oxygen-scavenging enzyme which produces hydrogen peroxide, had been previously identified as a mediator of metronidazole resistance^100^ and was found to be downregulated in all three isolates. Various nitroreductases, likewise identified previously^101–103^, were also downregulated. In contrast, multidrug resistance pump and metal *ABC transporter* genes were upregulated in all three resistant strains. The relative contribution of all of these factors to metronidazole resistance remains to be determined.

Importantly, treatment failures with metronidazole are not always caused by resistance as such. Treatment regimens can also strongly affect treatment outcome. It was demonstrated recently that a seven-day course with 500 mg metronidazole twice per day is clearly more effective than a single dose with 2 g^104^. In addition, it is necessary to test alternative treatments within the drug class of 5-nitroimidazoles, such as secnidazole^105^ or novel derivatives^106,107^.

Cross resistance, however, is often a problem with 5-nitroimidazole drugs, so completely different drugs have also been explored for anti-trichomonadal activity. Arguably, the most promising candidate is auranofin, a repurposed anti-rheumatic drug which was shown to be effective against a larger number of parasites^108^. Indeed, auranofin is also effective against *T*. *vaginalis* and can cure experimentally infected mice^109^. It was also successfully administered topically in mice on nanoparticles suspended in a hydrogel^110^. Thioredoxin reductase has been proposed to be the main target of auranofin, but this needs further confirmation as observations in another parasitic anaerobic flagellate, *Giardia lamblia*, contradict this notion^111^. Numerous other candidate drugs, including proteasome inhibitors such as carmaphycin-17^112^, zinc sulfate and zinc complexes^113,114^, bisbenzimidazole analogues^115^, boric acid^116^, and tetracycline^117^, were also evaluated. For a more complete overview of recent anti-trichomonadal drug research, a comprehensive review is available^118^.

As a concluding note, it is important to emphasize that assay conditions can be of very high importance when evaluating efficacies of established and novel anti-trichomonadal drugs. For example, cysteine, which is routinely used in growth media for *T*. *vaginalis*, has a highly protective effect against metronidazole and auranofin^119^.

## Concluding remarks

During the writing of this review, it became quickly apparent that the number of high-quality research articles in the *T*. *vaginalis* field has increased in the last five years as compared with the preceding quinquennial period^120^, a development which is also reflected in a larger number of references cited in this review. This is highly encouraging and indicates that *T*. *vaginalis* now receives more attention than before. There were substantial advances in our understanding of how *T*. *vaginalis* interacts with its host and, equally important, with the microbiome of which it is a part. As to the latter, the formation of consortia of protistologists, bacteriologists, and mycologists might even accelerate the pace of insightful discoveries in the future.
